# Nationwide patterns of hemorrhagic stroke among patients hospitalized with brain metastases: influence of primary cancer diagnosis and anticoagulation

**DOI:** 10.1038/s41598-020-67316-8

**Published:** 2020-06-22

**Authors:** Victor Lee, Vikram Jairam, James B. Yu, Henry S. Park

**Affiliations:** 1 Department of Therapeutic Radiology, Yale School of Medicine, New Haven, CT USA; 2 Cancer Outcomes, Public Policy, and Effectiveness Research (COPPER) Center, Yale School of Medicine, New Haven, CT USA

**Keywords:** CNS cancer, Cancer epidemiology

## Abstract

Brain metastases can contribute to a decreased quality of life for patients with cancer, often leading to malaise, neurologic dysfunction, or death. Intracerebral hemorrhage (ICH) is an especially feared complication in patients with brain metastases given the potential for significant morbidity and mortality. We aim to characterize patients with cancer and brain metastases admitted to hospitals nationwide and identify factors associated with ICH. The 2016 Healthcare Cost and Utilization Project Nationwide Inpatient Sample (HCUP-NIS) was queried for all patients with cancer hospitalized with a diagnosis of brain metastases. Admissions with a primary or secondary diagnosis of ICH were further identified. Baseline differences in demographic, clinical, socioeconomic, and hospital-related characteristics between patients with and without ICH were assessed by chi-square, Mann–Whitney U, and ANOVA testing. Multivariable logistic regression was used to identify factors associated with ICH. Weighted frequencies were used to create national estimates for all data analysis. In 2016, a total 145,225 hospitalizations were associated with brain metastases, of which 4,145 (2.85%) had a concurrent diagnosis of ICH. Patients with ICH were more likely to have a longer length of stay (median 5 days vs 4 days, *p* < 0.001) and a higher cost of stay (median $14,241.14 vs $10,472.54, *p * < 0.001). ICH was found to be positively associated with having a diagnosis of melanoma (odds ratio [OR] 5.01; 95% Confidence Interval [CI] 3.50–7.61) and kidney cancer (OR 2.50; 95% CI 1.69–3.72). Patients on long-term anticoagulation had a higher risk of ICH (OR 1.49; CI 1.15–1.91). Approximately 3% of patients hospitalized with brain metastases also had a diagnosis of ICH, which was significantly associated with longer length of stay and cost. Patients with melanoma, kidney cancer, and on long-term anticoagulation had a higher risk of ICH. Physicians should consider the risks of anticoagulation carefully for patients with brain metastases, especially those with melanoma and kidney cancer.

## Introduction

Brain metastases can contribute to a decreased quality of life for patients with cancer, often leading to death, neurologic dysfunction, headache, nausea, and fatigue^1,2^. Patients with cancer are also at risk for venous thromboembolism (VTE) and may be on anticoagulation. However, the possible complication of hemorrhagic stroke is especially feared in patients with brain metastases given the potential for significant morbidity and increased mortality^3^. Some studies have shown that there is no increased risk of intracerebral hemorrhage (ICH) with anticoagulation in patients with brain metastases. However, these conclusions do not take into account differences in the primary site of the tumor^4^, and so it is not clear if the benefits of anticoagulation on VTE outweigh the risks of ICH in all cases. Some studies have suggested that patients with brain metastases and primary cancers from melanoma, renal cell carcinoma, choriocarcinoma, thyroid carcinoma, and hepatocellular carcinoma are more likely to bleed spontaneously and may be at high risk of ICH with anticoagulation^5^. Despite the common occurrence of brain metastases and ICH, the frequency of their co-occurrence as well as risk factors associated with them are unknown.

Anticoagulation and primary cancer diagnosis may be factors associated with ICH as they are commonly associated with bleeding^6^. Other clinical risk factors for ICH may include older age or comorbidities like ischemic heart disease, diabetes mellitus, renal insufficiency, chronic liver disease, and alcohol addiction. However, it is unknown whether these factors apply for patients with brain metastases and how they should influence the decision to anticoagulate these patients who have VTE^7^.

To date, no study has examined ICH from brain metastases on a national level across several disease sites. Therefore, we decided to investigate the factors associated with ICH in patients with brain metastases. In this study, we aim to characterize patients with brain metastases admitted to hospitals nationwide and identify factors as well as treatments associated with ICH. An understanding of these factors may help physicians identify which patients with brain metastases can be anticoagulated versus managed with inferior vena cava (IVC) filters in order to balance the risks of VTE and ICH.

## Methods

This study used the National Inpatient Sample (NIS), which is the largest all-payer inpatient database in the United States, including over 7 million hospital stays every year from all participating states. The NIS is published by the Healthcare Cost and Utilization Project (HCUP) of the Agency for Healthcare Research and Quality. The NIS presents data from a 20% stratified sample of discharges throughout U.S. community hospitals. Each hospital visit is given a discharge weight so that a national estimate may be obtained. All diagnoses reported in our dataset from NIS were based on the International Classification of Diseases, Tenth Revision, Clinical Modification (ICD-10-CM) system. This study was granted an institutional review board exemption by the Yale Human Investigations Committee. Informed consent was also waived because the study was retrospective and data was deidentified.

**Figure 1:**
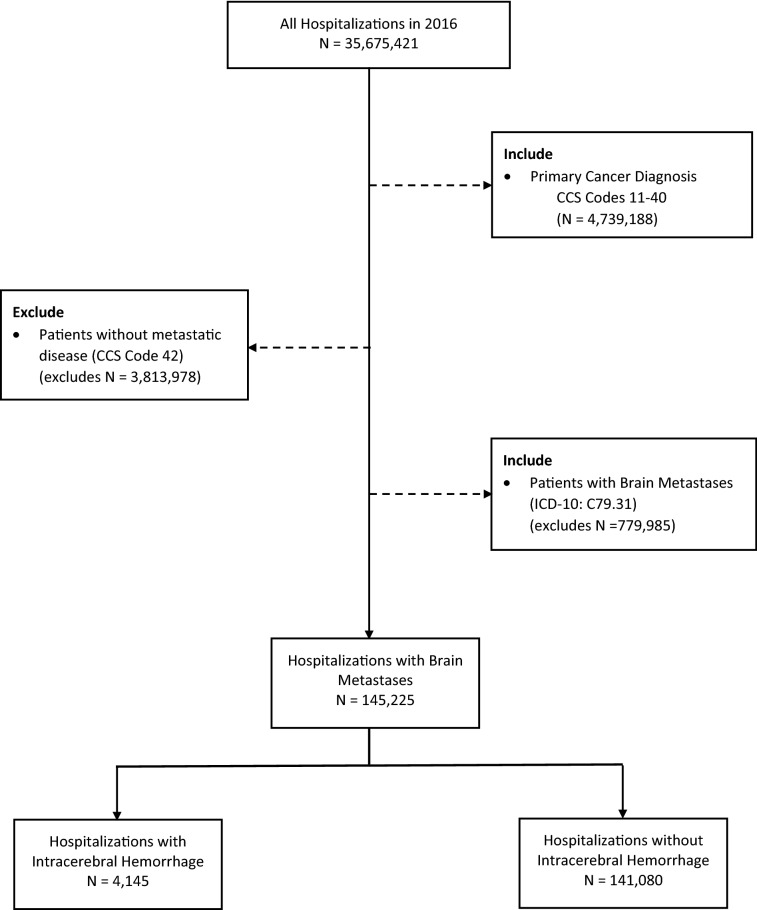
Consolidated Standards of Reporting Trials (CONSORT) diagram of cohort.

The NIS was queried in 2016 for all patients admitted with a known primary cancer diagnosis using the Clinical Classifications Software (CCS) codes 11–40. When patients had multiple cancers listed, we used the first listed cancer to classify the type of cancer the patient had. Patients without metastatic disease (CCS code 42) were excluded from analysis. Our cohort was then obtained by including patients with any listed diagnosis of brain metastasis (ICD-10-CM Code C79.31). Patients with a primary or secondary diagnosis of intracerebral hemorrhage were identified by ICD-10-CM Code I61.x. A Consolidated Standards of Reporting Trials (CONSORT) diagram detailing the methods used to identify our patient cohort is described in Fig. 1.

Hospital admissions were characterized by demographic factors (age, sex, race), socioeconomic factors (insurance type, income), clinical factors (comorbidities, cancer type), and hospital characteristics (region, location, size, teaching status). Long-term anticoagulation (ICD10 code Z79.01), hypertension (ICD10 code I10.x), and coagulopathy (ICD10 codes D65.x, D66.x, D67.x, D68.x, D69.1, D69.3, D69.4x, D69.5x, and D69.6) were identified a priori as comorbidities that could affect the risk of ICH and were thus also evaluated.

### Statistical analysis

Baseline differences in demographic, socioeconomic, and hospital characteristics between patients with and without ICH were assessed by chi-square, Mann–Whitney U, and ANOVA testing. Statistical significance for these analyses was set at *p* < 0.05. Multivariable logistic regression analyses were performed to identify factors associated with ICH. Models were adjusted for all demographic, socioeconomic, hospital factors, primary cancers, and comorbidities. Weighted frequencies were used to create national estimates for all data analysis. Hypothesis testing was two-sided. Data analysis was carried out using STATA v13.1 (StataCorp LP, College Station, TX).

## Results

### Hospitalizations for brain metastases

In 2016, there was a weighted total of 145,225 hospitalizations for brain metastases, of which 4,145 (2.9%) were associated with a primary or secondary diagnosis of ICH and 9,720 (6.7%) with long-term anticoagulation. The mean age of the overall cohort of patients with brain metastases was 62.9 years. The majority of patients were female (54.0%), were white (73.2%), used Medicare (49.2%), and were admitted to a larger hospital (60.4%) and a teaching hospital (74.0%). The median length of hospital stay was 4 days. The median cost per stay was $10,545.65.

### Characteristics of intracerebral hemorrhages

Baseline characteristics of patients with and without ICH are outlined in Table 1.

**Table 1:** Baseline characteristics of patients with or without intracerebral hemorrhage.

Variable	Weighted frequency (%)			*p* value
	All patients	No ICH	ICH	
Total number, N (weighted %)	145,225 (100)	141,080 (97.15)	4,145 (2.85)	
Age (mean, years)	62.89	62.86	63.91	0.030
Age category (years)				0.135
< 65	53.09	53.16	50.54	
≥ 65	46.91	46.84	49.46	
Sex				< 0.001
Male	45.98	45.67	56.35	
Female	54.02	54.33	43.65	
Race				0.460
White	73.17	73.14	74.18	
Black	13.30	13.36	11.21	
Hispanic	7.05	7.02	8.06	
Asian	3.17	3.16	3.40	
Other	3.31	3.31	3.15	
Median household income				0.004
$1–$41,999	27.65	27.72	25.28	
$42,000–$51,999	24.52	24.61	21.60	
$52,000–$67,999	24.75	24.74	25.15	
≥ $68,000	23.08	22.94	27.98	
Primary payer				0.023
Medicare	49.16	49.17	48.85	
Medicaid	13.85	13.94	10.52	
Private	31.78	31.74	33.37	
Self-pay	2.04	2.02	2.78	
No charge	0.17	0.17	0.24	
Other	3.00	2.96	4.23	
Hospital region				0.030
Midwest	23.26	23.36	19.9	
Northeast	21.44	21.41	22.44	
South	37.69	37.73	36.19	
West	17.61	17.49	21.47	
Hospital location				< 0.001
Rural	6.06	6.18	1.81	
Urban	93.94	93.82	98.19	
Hospital size				< 0.001
Small	13.73	13.94	6.63	
Medium	25.89	26.00	22.32	
Large	60.38	60.06	71.05	
Hospital teaching status				< 0.001
Non-teaching	26.03	26.29	17.13	
Teaching	73.97	73.71	82.87	
Primary cancer				< 0.001
Breast	12.69	12.84	7.72	
Lung	42.67	43.08	28.71	
Melanoma	4.70	4.37	15.80	
Kidney	3.43	3.36	5.79	
Thyroid	0.30	0.30	0.48	
Colon	1.90	1.90	1.93	
Other	34.31	34.15	39.57	
Comorbidities				
Long-term anticoagulation	6.69	6.62	9.29	0.003
Hypertension	45.20	45.07	49.46	0.011
Coagulopathy	10.65	10.62	11.70	0.308

**Figure 2:**
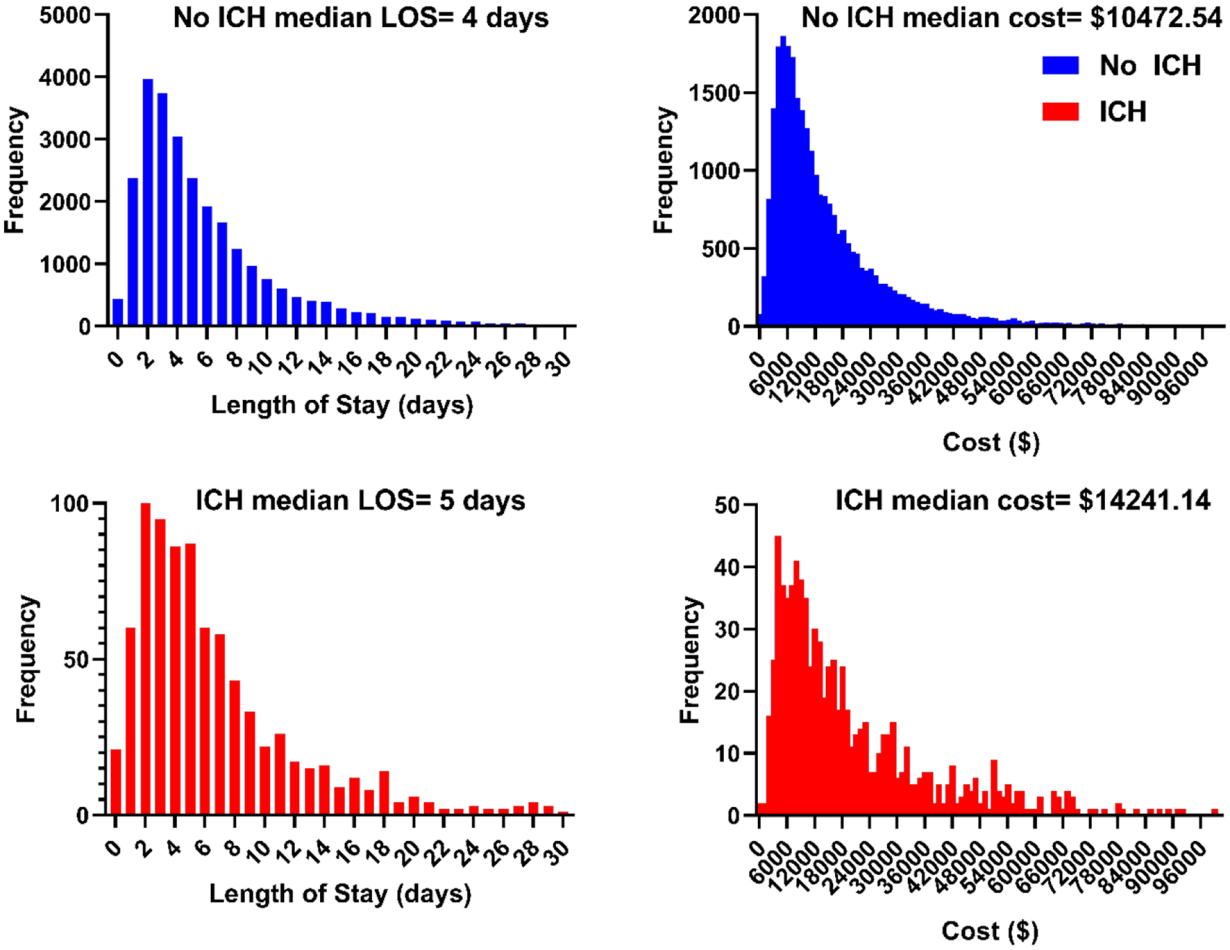
Histograms displaying length of stay and cost for patients with or without ICH.

Histograms of length of stay and cost for patients with and without ICH are displayed in Fig. 2. On unadjusted univariate analysis (all *p* < 0.05), patients with ICH were more likely to be female (54.0% vs 43.7%), present to an urban hospital (93.8% vs 98.2%), present to a large hospital (71.1% vs 60.1%), have a longer length of stay (median 5 days vs 4 days, *p* < 0.001), and have a higher cost of stay (median $14,241.14 vs $10,472.54, *p* < 0.001). The cancers with the highest proportion of hospitalizations with ICH were lung (43.1%), breast (12.8%), and melanoma (4.4%).

### Patient and hospital-related factors associated with intracerebral hemorrhage

On multivariable regression, multiple patient and hospital-related factors were associated with increased risk of ICH on admission. Patients who presented to a hospital in the West (odds ratio [OR] 1.37; 95% confidence interval [CI] 1.05–1.80), urban hospital (OR 2.31; 95% CI 1.30–4.09), medium-sized (OR 1.86; 95% CI 1.35–2.57) or large hospital (OR 2.53; 95% CI 1.89–3.39), and teaching hospital (OR 1.51; 95% CI 1.24–1.83) had an increased risk of ICH on admission. Female gender (OR 0.81; 95% CI 0.69–0.95) was found to be negatively associated with ICH (Table 2).

**Table 2:** Multivariable logistic regression for demographic, hospital-related factors, primary cancers, and comorbidities associated with intracerebral hemorrhage.

Variable	Odds ratio	95% CI	*p* value
Age category (years)			
< 65 (ref)			
≥ 65	1.13	0.92–1.40	0.244
Sex			
Male (ref)			
Female	0.81	0.69–0.95	0.008
Race			
White (ref)			
Black	1.02	0.80–1.31	0.847
Hispanic	1.08	0.78–1.48	0.649
Asian	1.12	0.75–1.67	0.575
Other	0.93	0.62–1.40	0.721
Median household income			
$1–$41,999 (ref)			
$42,000–$51,999	0.94	0.76–1.16	0.543
$52,000–$67,999	1.00	0.81–1.23	0.974
≥ $68,000	1.16	0.94–1.43	0.157
Primary payer			
Medicare (ref)			
Medicaid	0.89	0.65–1.23	0.491
Private	1.10	0.88–1.38	0.408
Self-pay	1.60	0.97–2.64	0.064
No charge	1.71	0.37–7.84	0.488
Other	1.57	1.06–2.31	0.024
Hospital region			
Midwest (ref)			
Northeast	1.19	0.90–1.57	0.215
South	1.20	0.95–1.52	0.136
West	1.37	1.05–1.80	0.021
Hospital location			
Rural (ref)			
Urban	2.31	1.30–4.09	0.004
Hospital size			
Small (ref)			
Medium	1.86	1.35–2.57	< 0.001
Large	2.53	1.89–3.39	< 0.001
Hospital teaching status			
Non-teaching hospital (ref)			
Teaching hospital	1.51	1.24–1.83	< 0.001
Primary cancer			
Breast (ref)			
Lung	1.02	0.75–1.40	0.889
Melanoma	5.01	3.51–7.16	< 0.001
Kidney	2.50	1.68–3.72	< 0.001
Thyroid	1.80	0.54–6.00	0.338
Colon	1.57	0.88–2.78	0.126
Other	1.63	1.21–2.20	0.001
Long-term anti-coagulation			
No long-term anticoagulation (ref)			
Long-term anticoagulation	1.49	1.15–1.91	0.002
Hypertension			
No hypertension (ref)			
Hypertension	1.34	0.82–2.19	0.236
Coagulopathy			
No coagulopathy (ref)			
Coagulopathy	2.10	1.18–3.73	0.012

### Association between primary cancer type and comorbidities with intracerebral hemorrhage

Among cancer types, melanoma (OR 5.01; 95% CI 3.51–7.16) and kidney cancer (OR 2.50; 95% CI 1.68–3.72) were positively associated with ICH. Meanwhile among comorbidities, long-term anticoagulation (OR 1.49; CI 1.15–1.91) was associated with ICH (Table 2). Among patients with brain metastases hospitalized with long-term anticoagulation, a diagnosis of melanoma (OR 4.98; CI 1.73–14.33) and a comorbid coagulopathy (OR 2.05; CI 1.14–3.69) were significantly associated with ICH (Table 3).

**Table 3:** Multivariable logistic regression for demographic, hospital-related factors, primary cancers, and comorbidities associated with intracerebral hemorrhage among the subset of patients undergoing long-term anticoagulation.

Variable	Odds ratio	95% CI	*p* value
Age category (years)			
< 65 (ref)			
≥ 65	1.53	0.80–2.93	0.199
Sex			
Male (ref)			
Female	0.89	0.52–1.52	0.673
Race			
White (ref)			
Black	0.74	0.31–1.79	0.506
Hispanic	0.38	0.09–1.73	0.211
Asian	2.02	0.63–6.47	0.238
Other	N/A	N/A	N/A
Median household income			
$1–$41,999 (ref)			
$42,000–$51,999	0.76	0.33–1.74	0.520
$52,000–$67,999	1.02	0.52–1.98	0.961
≥ $68,000	1.03	0.49–2.14	0.942
Primary payer			
Medicare (ref)			
Medicaid	1.16	0.36–3.72	0.797
Private	1.78	0.89–3.56	0.101
Self-pay	1.14	0.12–11.00	0.908
No charge	N/A	N/A	N/A
Other	2.86	0.80–10.16	0.104
Hospital region			
Midwest (ref)			
Northeast	1.73	0.83–3.61	0.143
South	1.35	0.65–2.80	0.425
West	1.96	0.92–4.20	0.082
Hospital location			
Rural (ref)			
Urban	1.04	0.21–5.13	0.962
Hospital size			
Small (ref)			
Medium	1.48	0.57–3.82	0.423
Large	2.20	0.97–5.02	0.060
Hospital teaching status			
Non-teaching hospital (ref)			
Teaching hospital	1.72	0.87–3.42	0.121
Primary cancer			
Breast (ref)			
Lung	0.70	0.28–1.78	0.457
Melanoma	4.98	1.73–14.33	0.003
Kidney	1.04	0.20–5.37	0.963
Thyroid	N/A	N/A	N/A
Colon	0.72	0.07–7.30	0.779
Other	1.55	0.64–3.75	0.334
Hypertension			
No hypertension (ref)			
Hypertension	1.37	0.84–2.24	0.201
Coagulopathy			
No coagulopathy (ref)			
Coagulopathy	2.05	1.14–3.69	0.016

## Discussion

This study provided a national analysis of risk factors associated with ICH in patients who presented with brain metastases in the inpatient setting. We found that melanoma, kidney cancer, and long-term anticoagulation were positively associated with ICH. Some studies have suggested these primary cancers may be associated with ICH, but long-term anticoagulation has not previously been shown to be a risk factor for ICH in patients with brain metastases. In a retrospective study of 905 patients with brain tumors, those with metastatic melanoma had the highest rate of hemorrhage at 50%^8^. In another retrospective study, patients with brain metastases from renal cell carcinoma and melanoma were more likely to develop ICH but it was not clear whether anticoagulation was safe in this population^5^. An additional retrospective study also noted that patients with brain metastases from melanoma or renal cell carcinoma were more likely to develop ICH but suggested that long-term anticoagulation may not be associated with ICH^9^.

Potential mechanisms have been suggested regarding a possible link between the expression of molecular markers and tendency of certain primary tumors to metastasize and cause hemorrhage. Overexpression of VEGF and MMP may contribute to the development of ICH through the rapid growth and destruction of peritumoral vessels^10^. VEGF expression may promote melanoma growth by stimulating angiogenesis^11^.

Besides tumor-related factors, our analysis found that there were other patient-related and facility-related factors associated with ICH. Male sex was found to be associated with ICH in patients with brain metastases. Studies have found that male gender was significantly associated with ICH in a non-cancer setting^12,13,14^. This effect may be due to several biological and social causes of the disease. For example, estrogen has been shown to be cardioprotective in several ways^15^ whereas androgens stimulate the progression of atherosclerosis^16^. Larger hospital size and teaching hospitals were also found to be significantly associated with ICH. This may be due to the possibility that the most complex cases may be more often referred to large academic institutions with the resources to manage the high acuity of patients with ICH and brain metastases.

Hypertension is a well-known risk factor for ICH and strokes in general^17,18,19^. In a retrospective cohort study, Schmidt et al. found that surgical treatment and renal insufficiency were associated with ICH. Additionally, antihypertensive treatment was associated with a reduced risk of ICH^7^. In our study, hypertension was not significantly associated with ICH. However, we were unable to control for whether the patients were on antihypertensive medications and how well hypertension was controlled, and so further investigation will be needed to determine the interaction of uncontrolled hypertension with other risk factors in our study with regards to ICH in brain metastases.

The main limitations of this study are those that are inherent to retrospective studies that utilize national databases. First, this data is limited to a single year of data so it was not possible to determine trends. This was done intentionally as metastasis of the brain could only be determined with ICD-10 coding rather than the less specific ICD-9 coding, which is unable to distinguish between brain and spinal cord metastasis. Second, the NIS does not code for patient-level data, and so multiple hospitalizations may have occurred for the same patient. Third, temporal information is not provided in this database and it is unclear whether the patients developed ICH or brain metastasis first. Fourth, ICD coding lacks details regarding the agent and timing of “long-term anticoagulation,” as well as granular breakdown of types of “kidney cancer” to determine the number harboring renal cell carcinoma. Finally, the NIS represents the hospitalized patient population and is not necessarily generalizable to the non-hospitalized population.

In conclusion, ICH can be a significant complication of patients with brain metastases, especially in those with melanoma, kidney cancer, and a history of long-term anticoagulation. When examining the subset of patients who were on long-term anticoagulation, melanoma was still highly associated with ICH. While patients with brain metastases are often in a hypercoagulable state, the risks and benefits of anticoagulation must be weighed carefully, especially for those with melanoma or kidney cancer. These patients may also benefit from more intensive monitoring and follow-up. Further research is needed in order to determine the optimal guidelines for anticoagulation management in patients with brain metastases.
